# Antioxidant Activity, Antitumor Effect, and Antiaging Property of Proanthocyanidins Extracted from *Kunlun Chrysanthemum* Flowers

**DOI:** 10.1155/2015/983484

**Published:** 2015-01-01

**Authors:** Siqun Jing, Xiaoming Zhang, Liang-Jun Yan

**Affiliations:** ^1^State Key Laboratory of Food Science and Technology, School of Food Science and Technology, Jiangnan University, Lihu Road 1800, Wuxi, Jiangsu 214122, China; ^2^College of Life Sciences and Technology, Xinjiang University, Shengli Road 14, Urumqi, Xinjiang 830046, China; ^3^Department of Pharmaceutical Sciences, UNT System College of Pharmacy, University of North Texas Health Science Center, 3500 Camp Bowie Boulevard, Fort Worth, TX 76107, USA

## Abstract

The objective of the present study was to evaluate the antioxidant activity, antitumor effect, and antiaging property of proanthocyanidins from *Kunlun Chrysanthemum* flowers (PKCF) grown in Xinjiang. In vitro antioxidant experiments results showed that the total antioxidant activity and the scavenging capacity of hydroxyl radicals (^•^OH) and 1,1-diphenyl-2-picrylhydrazyl (DPPH^•^) radicals increased in a concentration-dependent manner and were stronger than those of vitamin C. To investigate the antioxidant activity of PKCF in vivo, we used serum, liver, and kidney from mouse for the measurement of superoxide dismutase (SOD), malondialdehyde (MDA), and total antioxidant capacity (T-AOC). Results indicated that PKCF had antioxidative effect in vivo which significantly improved the activity of SOD and T-AOC and decreased MDA content. To investigate the antitumor activity of PKCF, we used H22 cells, HeLa cells, and Eca-109 cells with Vero cells as control. Inhibition ratio and IC_50_ values were measured by 3-(4,5-dimethylthiazol-2-yl)-2,5-diphenyltetrazolium bromide (MTT) assay; PKCF showed great inhibitory activity on H22 cells and HeLa cells. We also used fruit flies as a model for analyzing the anti-aging property of PKCF. Results showed that PKCF has antiaging effect on *Drosophila*. Results of the present study demonstrated that PKCF could be a promising agent that may find applications in health care, medicine, and cosmetics.

## 1. Introduction

The scientific name of* Kunlun Chrysanthemum* from Xinjiang, China, is* Coreopsis tinctoria*, annual herb, family Compositae [1].* Kunlun Chrysanthemum* naturally grows in the Kunlun Mountains region at an elevation of 3000 meters in Hetian region of Xinjiang; its Uighur name is “guriqiayi” [2]. It is a rare alpine wild plant with a unique effect and high medicinal value [3]. Currently, the main product of* Kunlun Chrysanthemum* on market is dry tea, with almost no refinery processed products. As people have come to realize the health benefits of* Kunlun Chrysanthemum*, research on the active ingredients of* Kunlun Chrysanthemum* gradually becomes the focus of numerous studies [4–7]. However, due to its limited origin that is highly regional, it is not widely available. Hence, in-depth research remains to be undertaken. As a high-grade tea its market prospect is promising and further may yield high medical value. Indeed, it is widely known that the flowers of* Chrysanthemum morifolium* Ramat, which is widely used as a food supplement or herb tea, are considered as a healthy food supplement by many consumers [8].* Chrysanthemum morifolium* extract has also been demonstrated to be safe as a traditional herbal medicine [9, 10]. Therefore we reasoned that PKCF would have special biological activities.

Proanthocyanidins (PC) are a generic term of a broad class of polyphenol compounds polymerized by flavan-3-ol or flavan-3,4-diol [11]. PC are internationally recognized as the most effective natural antioxidants against free radicals [12, 13]. Furthermore, PC have been shown to prevent cardiovascular diseases [14] and to exhibit antitumor [15] and antihypertensive effects [16] and to prevent radiation, mutation, and vision degeneration and skin disorders. Additionally, PC have the advantages of improving human microcirculation. Therefore, research on PC has become increasingly important because of their various strong biological effects. It should be noted that, as a secondary metabolite from plants, PC are widely distributed in seeds, leaves, fruit, flowers and skin, shell, and so forth of various plants. For example, PC have been mostly extracted from grape seed worldwide, followed by the bark of pine tree [17, 18]. To our knowledge, there is no information published about antioxidant activity and antitumor effect of PKCF. Additionally, we have not found any report related to antiaging effect of PKCF.

In the present study, the antitumor effect and antiaging of PKCF were evaluated by MTT assay and* Drosophila melanogaster *survival test [19]. The antioxidant activities of PKCF were also investigated by antioxidant experiments in vitro and in vivo. This research may provide scientific evidence for the further application of PKCF in food or pharmacology.

## 2. Material and Methods

### 2.1. Materials

Dried* Kunlun Chrysanthemum* flowers were provided by Xinjiang Hetian Shamo Meigui Co., Ltd. PKCF with purity of63.76 ± 0.34 % (w/w) was obtained through extraction by ultrasonic assisted method and purified by AB-8 macroporous adsorption resin. Standard PC (assessed by UV ≥95%) used in vitro studies was purchased from Shanghai Yuanye Biotechnology Co., Ltd.

### 2.2. Animals

Six-to-eight-week-old Kunmin female mice (20 ± 2 g) 2011-0003/SCXK (Xin) were obtained from Xinjiang Laboratory Medical University Breeding Research Center. Composition of the mouse diet was dregs of beans, meat and bone meal, corn flour, salt, multivitamins, and sunflower cake.

Wild-type strain* Drosophila melanogaster *(Oregon R+) was kindly provided by Genetics Laboratory of College of Life Sciences and Technology, Xinjiang University (Urumqi, China).* Drosophila melanogaster* were cultured in bottles with standard* Drosophila* medium at a temperature of 25 ± 1°C and a relative humidity of 50~60%.

### 2.3. *In Vitro* Antioxidant Potential of PKCF

PC was found to be the most powerful free radical scavenger by far and has a very strong activity in vivo [20]. PC has been proven to be 30~50 times more effective than vitamins C and E in terms of free radical scavenging [21]. To study the potential antioxidant health-protecting effects of natural products, however, a single in vitro chemical method may not be enough to evaluate and compare their antioxidant properties. Among the free radical species, DPPH^•^ is more stable and has the ability to become a stable diamagnetic molecule by accepting an electron or hydrogen. Therefore, it has been widely used to evaluate the antioxidative activity of natural antioxidants [22]. In addition, ^•^OH and its subsequent radicals are also considered as the most harmful ROS and are responsible for the oxidative injury of biomolecules [23]. Therefore, in the present study, the scavenging activities against DPPH^•^, ^•^OH, and Fe^2+^ chelating activity were selected to evaluate the antioxidant activity of PKCF in vitro. All experiments were carried out in triplicate and at least on two separate occasions and with ascorbic acid (Vc) as a positive control whose concentration was the same as that of PKCF in each experiment. In vitro, the concentrations of PKCF used were determined by preliminary experiments in which the absorbance of PKCF fell in a linear range of 0.2 and 0.7.

#### 2.3.1. Assay of Hydroxyl Radical Scavenging Activity

The hydroxyl radical scavenging activity of PKCF was measured by the method of Xiao et al. [24]. Briefly, 4 mL of sodium phosphate buffer (pH 7.4) was added to a test tube and mixed with 1.5 mL of 5 mmol/L phenanthroline solution. Next, 1 mL of 7.5 mmol/L FeSO_4_ solution and 1 mL of the nine different sample solutions of PKCF (0.01, 0.02, 0.03, 0.04, 0.05, 0.06, 0.1, 0.2, and 0.3 mg/mL) were added to the solution.

Finally, 1.5 mL of double distilled water and 1.0 mL of 10 g kg^−1^ H_2_O_2_ solution were added. The absorbance of the final solutions was measured at 536 nm with a UV-visible spectrophotometer after incubation at 37°C for 60 min. Antioxidant value was expressed as IC_50_, which represented the concentration of PKCF that caused 50% inhibition of hydroxyl radical formation.

#### 2.3.2. Assay of DPPH Radical Scavenging Activity

The DPPH radical scavenging activity of PKCF was measured according to the reported method of Brand-Williams et al. [25] with some modifications. Briefly, 2 mL of PKCF solution with different concentrations (0.01~0.2 mg/mL) was added to 2 mL DPPH solution. After shaking vigorously, the mixture was incubated at 25°C in the dark for 30 min. The absorbance was measured at 517 nm by ultraviolet-visible spectrometer. A lower absorbance of the reaction mixture would indicate a higher free radical scavenging activity. Moreover, in order to compare the radical scavenging efficiency of the extracts, IC_50_ value showing the concentration of PKCF that caused 50% scavenging of DPPH radical was calculated by use of invariant linear regression equation.

#### 2.3.3. Assay of Reducing Power

The reducing power of all the fractions and ascorbic acid was performed as previously described by Pan et al. [26] with a slight modification. In this assay, the antioxidants present in the test solution can reduce the Fe^3+^/ferricyanide complex to the ferrous form by donating an electron. The color of the test solution then changes from yellow to different shades of green and blue, which depends on the reducing power of PKCF. Different concentrations (0.01~0.05 g/mL) of sample solutions were prepared from the stock solution in DMSO. An aliquot of the sample solutions (1 mL) was combined with 2.5 mL of 0.2 M phosphate buffer (pH 6.6) and 2.5 mL of 1% (w/v) potassium ferricyanide. After the mixture was incubated at 50°C for 20 min, a portion (2.5 mL) of 10% trichloroacetic acid was added to the mixture to stop the reaction. Then the mixture was mixed with 2.5 mL distilled water and 2.5 mL of 0.1% ferric chloride solution. After standing for 10 min, the absorbance was measured at 700 nm with a UV-visible spectrophotometer against a blank. The control contained all reagents except PKCF. Higher absorbance indicates higher reducing power. The absorbance A was measured at 700 nm.

### 2.4. *In Vivo* Antioxidant Potential of PKCF

Antioxidant activity in vivo of PKCF was carried out by the method based on Jing et al. [27] with a few modifications. The concentrations of PKCF used were based on data reported in the literature in conjunction with purity and dosage used in mice. The mice were allowed to acclimatise to the laboratory for a week. They were then divided into five groups: normal control group (NC), low-dose group (LD, 50 mg kg^−1^ bw^−1^ d^−1^, where bw is body weight), middle-dose group (MD, 100 mg kg^−1^ bw^−1^ d^−1^), high-dose group (HD, 200 mg kg^−1^ bw^−1^ d^−1^), and positive control group (NC^+^ vitamin C 800 mg kg^−1^ bw^−1^ d^−1^). The LD, MD, and HD mice groups were fed PKCF once a day for 28 days, while the NC^+^ group was fed vitamin C once a day and the NC group was given the same amount of physiological saline. Gastric volume was of 0.2 mL. The food and water intake by the animals were monitored daily and the body weight was measured weekly. After 4 weeks, the mice were fasted overnight without limiting the water. Blood was then taken from the mice's eyes and centrifuged at 3000 ×g for 15 min for supernatant analysis. The mice were then killed, after which their organs were dissected and the connective tissue was immediately weighed. Physiological saline was subsequently added to make 10% concentration of liver homogenate at 4°C and then centrifuged at 3500 ×g for 5 min. The supernatant was then removed and refrigerated at −20°C. The value of superoxide dismutase (SOD), malondialdehyde (MDA), and total antioxidant capacity (T-AOC) from serum, liver, and kidney was determined by using commercial reagent kits according to the instruction manuals.

### 2.5. Assay of* In Vitro* Antitumor Activity

Cervical cancer HeLa cells, esophagus cancer Eca-109 cells, Vero cells, and mouse ascites hepatomas H22 cells were obtained from Xinjiang University Xinjiang Biological Resources Gene Engineering Key Laboratory (Urumqi, China). In vitro antitumor activity of PKCF was determined using three kinds of tumor cells (HeLa, Eca-109, and H22), together with Vero cells as normal cell controls. The proliferation of cells mentioned above was determined using the colorimetric MTT assay described by Mosmann [28]. Briefly, various cells were seeded in 96-well flat plates and allowed to adhere for 24 h at 37°C with 5% CO_2_ in the atmosphere. The cultures were washed and treated with a serial concentration of PKCF (15 *μ*g/mL, 31.25 *μ*g/mL, 62.5 *μ*g/mL, 125 *μ*g/mL, 250 *μ*g/mL, 500 *μ*g/mL, and 1000 *μ*g/mL). MTT (5 mg/mL; Sigma-Aldrich, MO, USA) was then added 12 h, 24 h, 48 h, or 72 h later. After the plates were incubated at 37°C for 4 h, at the end of the treatment, the incubation medium was discarded, and the formed crystals were dissolved in 200 *μ*L of DMSO. MTT reduction was quantified by measuring the light absorbance of each well at 570 nm to evaluate the proliferation of cancer cells while the reference wavelength was 650 nm. All experiments were performed in triplicate. OD value of each well was measured [29].

### 2.6. Antiaging Experiments

#### 2.6.1. Preparation of Cornmeal Medium Applied to* Drosophila melanogaster*

The cornmeal medium used in this study as culture medium for* Drosophila melanogaster* contained cornmeal, sugar, dry yeast, and agar and was prepared according to a previously published protocol [30] with the formula (g/100 mL) as follows: cornmeal 11.33 g, agar 0.7 g, and sugar 8 g, mixing all the ingredients in the 100 mL distilled water to boil for 3 min, then adding 0.53 g yeast powder and 0.33 mL acid immediately, and then pouring into sterile tubes about 20 to 30 mm of thickness, staying cool for experiments.

#### 2.6.2. The Dosage Choice of Test Substance

According to the recommended intake dosage of 0.0033 g·kg^−1^ bw^−1^ d^−1^ for human recommended by China Food and Drug Administration (CFDA), the experimental middle concentration was calculated to be of 0.0067% [31]. Therefore, four dose groups were formed through setting 1~2 concentration groups below and above the middle concentration of 0.067%, which were low-dose groups (0.0022%), medial-dose groups (0.0067%), and high-dose groups (0.0201%, 0.0603%).

#### 2.6.3. *Drosophila melanogaster* Survival Test

*Drosophila melanogaster* were allowed to lay eggs on the cornmeal medium. After approximately two weeks, posteclosion adults were collected and transferred to new bottles of 2.5 cm × 20 cm tubers with fresh medium within 8 h. 600 flies were applied and randomly divided into 5 groups with each group of 120 (half male and half female) and then cultivated under conditions of 25 ± 1°C and relative humidity 45~75%. Flies were then transferred to fresh medium every 4 days. Survival activity was observed and the number of dead flies was counted daily and until all fruit flies were dead. The day on which 50% flies died was used to calculate the average lifespan and the average maximum lifespan [32].

#### 2.6.4. Assay of Biochemical Index* In Vivo* of* Drosophila melanogaster*

Strategies designed to reduce oxidative damage have been shown to extend lifespan of an organism. Here we used* Drosophila melanogaster* as a model to evaluate whether and how supplementation of PKCF in the flies' food promotes longevity. Briefly, flies were fed according to the methods described above until they were 30 days old followed by antiaging test as described previously [33]. Each group was weighed and homogenized with cold saline to make 1% tissue homogenate and centrifuged (3000 ×g for 10~15 min) to get supernatant for further studies. The median lethal time (LT_50_), average lifespan, average maximum life span, average life extension rate, and average maximum life extension rate of five samples from each group were measured, and SOD activity and MDA content were analyzed according to the instructions given by the manufacturer.

### 2.7. Statistical Analysis

All data were shown as means ± SD (standard deviation) of three independent measurements. Statistical calculations were carried out by SPSS version 17.0 (SPSS Inc., Chicago, USA). ANOVA was applied for determining differences between the results of samples. Values of *P* < 0.05 were considered significantly different.

## 3. Results and Discussion

### 3.1. Antioxidant Activity* In Vitro*

#### 3.1.1. Scavenging Activity of Hydroxyl Radicals

**Figure 1:**
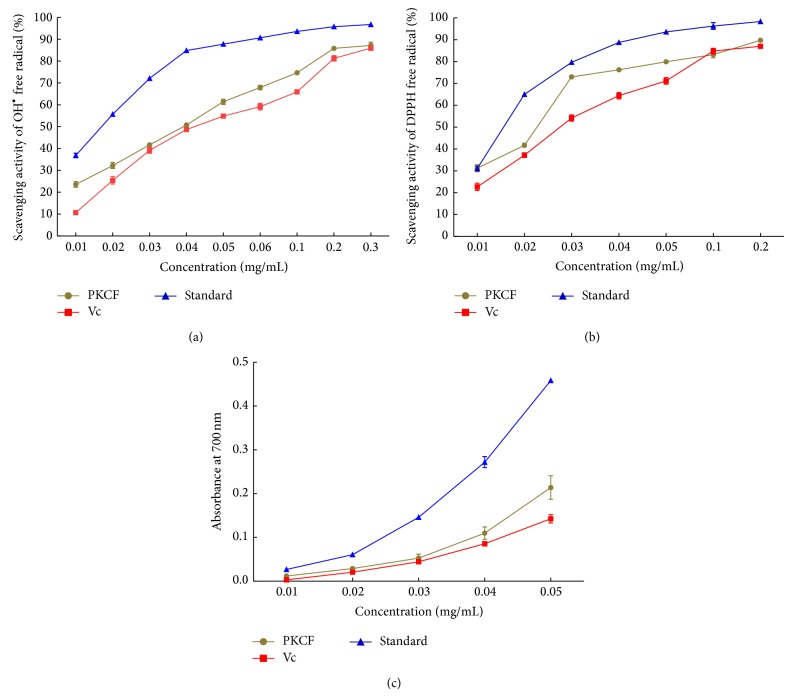
Antioxidant activity of PKCF in vitro: (a) scavenging activity of hydroxyl radicals, (b) scavenging activity of DPPH radicals, and (c) total antioxidant capacity. All compared with those of vitamin C and standard of proanthocyanidins (PC).

PKCF was found to have the ability to scavenge hydroxyl radicals at concentrations between 0.01 mg/mL and 0.3 mg/mL. As shown in Figure 1(a), the scavenging activity of PKCF on hydroxyl radicals was in a concentration-dependent manner. The scavenging capacity of PKCF on hydroxyl groups was higher than the positive control vitamin C while lower than PC. The scavenging ability of PKCF was stronger at higher concentration with a value of 88.3% at the concentration of 0.3 mg/mL while that of Vc and Pc at the same concentration (0.3 mg/ml) were 86.13% and 97.2%, respectively, but these values declined quickly with lower concentrations. For hydroxyl radical scavenging of PKCF, there were two models of antioxidation mechanisms: one suppresses the generation of hydroxyl radical and the other scavenges the hydroxyl radicals produced. The IC_50_ of PKCF for scavenging hydroxyl radical was higher (0.03560 ± 0.06335 mg/mL) than that of vitamin C (0.04793 ± 0.07487 mg/mL) and lower than that of PC (0.01519 ± 0.1182 mg/mL). Thus, PKCF can be considered as an effective scavenger of hydroxyl radical.

#### 3.1.2. DPPH Scavenging Activity

The change of concentration of PKCF was monitored to investigate the antioxidant effect of PKCF through the DPPH scavenging ability test. As shown in Figure 1(b), the scavenging activity of PKCF on hydroxyl radicals was in a concentration-dependent manner. The scavenging ability of PKCF was stronger at higher concentration with a value of 89.28% at the concentration of 0.2 mg/mL while that of Vc and Pc at the same concentration (0.2 mg/ml) were 86.43% and 98.6%, respectively, indicating that PC has stronger DPPH radical scavenging activity. In general, PKCF showed higher antioxidant activity (IC_50_ = 0.01851 ± 0.2434 mg/mL) than vitamin C (IC_50_ = 0.02693 ± 0.09211 mg/mL) but lower activity than PC (0.01464 ± 0.06483 mg/mL). The results indicate that PKCF had a strong DPPH radical scavenging activity. The DPPH scavenging activities could be attributed to their hydrogen donating abilities. Hence, the mechanism may be due to the supply of hydrogen by PKCF, which combines with radicals and forms a stable radical to terminate the radical chain reaction [34].

#### 3.1.3. Total Antioxidant Capacity

Reducing power is an important indicator of antioxidants that provide H^•^. By providing H^•^, antioxidants can transform free radicals into stable molecules, thereby decreasing the damaging effects of free radicals. The results presented in Figure 1(c) show that the total antioxidant activity increased as the concentration of PKCF increased. However, at a PKCF concentration of 0.02 mg/mL, the total oxidant capacity increased slowly. The scavenging ability of PKCF was stronger at higher concentrations, with absorbance value of 0.233 at the concentration of 0.05 mg/mL, while the values for vitamin C and PC were 0.149 and 0.456, respectively. Therefore, reducing powers of the three tested samples were as follows: vitamin C < PKCF< PC.

In summary, we presented our findings that the antioxidant activity of PKCF in vitro was stronger than that of vitamin C.

### 3.2. Antioxidant Activity* In Vivo*

#### 3.2.1. Influence of Sample on Weight, Spleen Index, and Thymus Index of Mice

To investigate whether PKCF has any effects on mice, we further weighed and analyzed spleen index and thymus index of mice. Results were shown in Tables 1 and 2.

The effect of PKCF on the body weight gain was examined in Table 1. The final body weight of all groups showed no significant differences (*P* > 0.05) in the food intake between control and sample groups; the results indicated that the dosage of PKCF had little effect on the body weight increase of mice.

As shown in Table 2, there was no significant difference between experimental groups with LD, MD, and HD and control group (*P* < 0.05) in spleen index and thymus index, and the effect of PKCF is not dose responsive, which suggested that feeding mice with PKCF could not induce any physical damage to mice.

#### 3.2.2. Values of SOD in the Tissues and Serum of Mice

Superoxide dismutase (SOD) is the major enzyme capable of removing highly reactive and toxic superoxide radicals that are generated during metabolic processes [35]. As shown in Table 3, our results revealed that ingestion of PKCF had a significant effect on SOD activity in serum and tissue of mice (*P* < 0.05). Specifically, MD groups and HD groups differed significantly and increased in serum when compared with control group, while NC groups and LD groups did not increase (*P* < 0.01 and *P* < 0.05). On the other hand, three different doses could significantly improve liver and kidney SOD activities (*P* < 0.01 and *P* < 0.05). There were also significant differences in SOD activity between positive control group (vitamin C) and the control group (*P* < 0.05), and the effects of positive control (vitamin C) were equivalent to that of the HD groups. Thus, the results showed that PKCF could improve the SOD activity in mice and reflected an obvious dose-dependent effect.

#### 3.2.3. Values of MDA in the Tissues and Serum of Mice

The MDA content (Table 4) differed significantly between the NC^+^ and NC groups (*P* < 0.05) in serum, liver, and kidney, and this phenomenon indicated that the presence of vitamin C in the NC^+^ diet could reduce the MDA content. At the same time, the effect of MD groups and HD groups on MDA content of serum and liver tissue of mice differed significantly (*P* < 0.01 & *P* < 0.05). These observations showed that PKCF could decrease the MDA content in mice in a dose-dependent manner.

#### 3.2.4. Values of T-AOC of Tissues and Serum from Mice

As shown in Table 5, the T-AOC of MD and HD groups was significantly or extremely significantly higher than that of normal control group except for LD group and NC^+^ group. These results indicated that PCKC could improve total antioxidant capacity in mice and showed a dose-dependent relationship. Results suggest that PKCF can significantly enhance the SOD activity in test animals and further decrease the MDA level and improve T-AOC in vivo through antioxidant enzymes. Furthermore, it scavenges a large number of free radicals produced by metabolism [36].

In summary, the results of in vitro and in vivo experiments showed that PKCF had antioxidant activity and scavenging activity on hydroxyl radical and DPPH radical and total antioxidant activity were stronger than those of vitamin C. In vivo, PKCF could significantly improve the content of SOD without increasing the MDA levels significantly. Overall, PKCF showed considerable antioxidant and radical scavenging activity.

### 3.3. *In Vitro* Antitumor Activity of PKCF

Eca-109 cells, HeLa cells, and H22 cells were incubated with different concentrations (15 *μ*g/mL, 31.25 *μ*g/mL, 62.5 *μ*g/mL, 125 *μ*g/mL, 250 *μ*g/mL, 500 *μ*g/mL, and 1000 *μ*g/mL) of PKCF for a certain time (24, 48, and 72 h) and then were measured by MTT assay.

#### 3.3.1. MTT Assay for Cell Proliferation Inhibition

**Figure 2:**
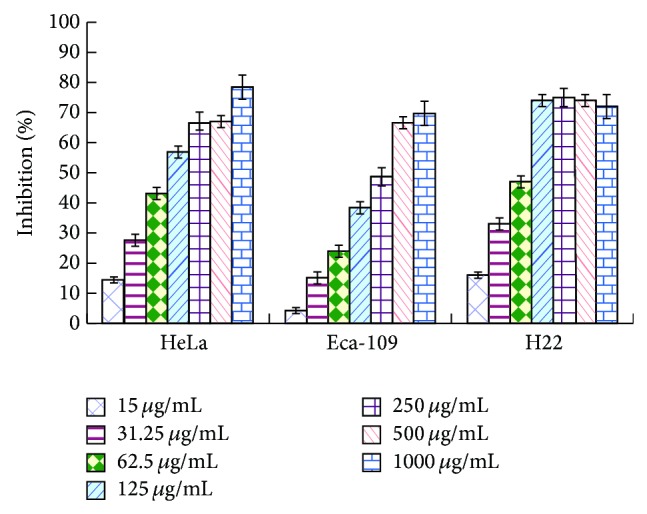
The curve of PKCF against HeLa, Eca-109, and H22 cells proliferation for 72 h.

As shown in Figure 2, the inhibition rate of PKCF on HeLa and H22 cell showed significant difference compared with negative control group (*P* < 0.01) and displayed a satisfied dose and time-dependent relationship. PKCF could inhibit the proliferation of Eca-109, HeLa, and H22 with average IC_50_ values of 260.4 ± 0.06887 *μ*g/mL, 113.3 ± 0.08062 *μ*g/mL, and 70.96 ± 0.05409 *μ*g/mL, respectively, and showed stronger inhibition on H22 proliferation.

#### 3.3.2. Effect on Proliferation of HeLa Cells

**Figure 3:**
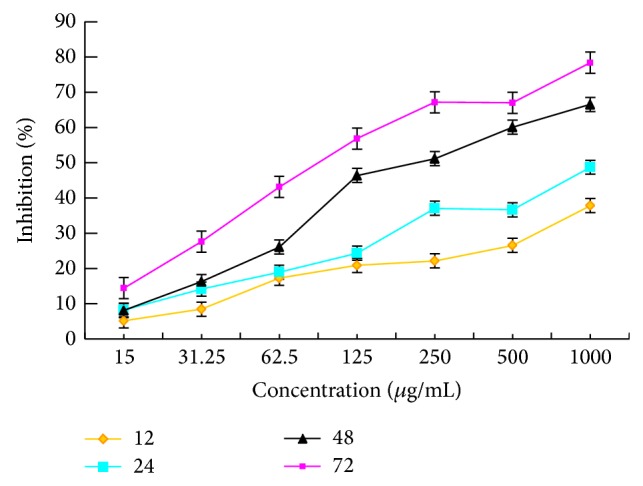
Time curve of different concentrations of PKCF against HeLa cells proliferation.

As shown in Figure 3, PKCF could specifically inhibit the growth of HeLa cells in a dose- and time-dependent manner with the IC_50_ values of 3315 ± 0.06018 *μ*g/mL, 1106 ± 0.04979 *μ*g/mL, 259.7 ± 0.08211 *μ*g/mL, and 113.3 ± 0.08062 *μ*g/mL, respectively, after HeLa cells treatment for a certain time of 12 h, 24 h, 48 h, and 72 h. At the concentration of 1000 *μ*g/mL, the inhibitory rate of HeLa cells was 78.42%. The results of the study are similar to that of Okamoto et al. [37] and Apostolou et al. [38]. PKCF can significantly inhibit the proliferation of HeLa.

#### 3.3.3. Effect on Proliferation of Eca-109 Cells

**Figure 4:**
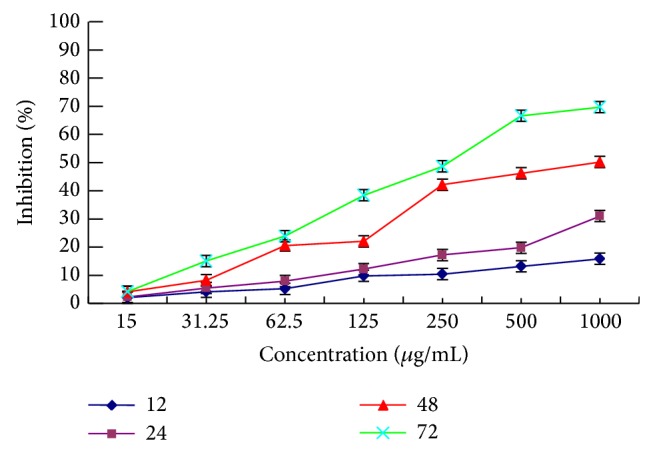
Time curve of different concentrations of PKCF against Eca-109 cells proliferation.

As shown in Figure 4, PKCF inhibited the growth of Eca-109 in a dose- and time-dependent manner with the IC_50_ values of 51646 ± 0.05027 *μ*g/mL, 4208 ± 0.04590 *μ*g/mL, 704.6 ± 0.09879 *μ*g/mL, and 260.4 ± 0.06887 *μ*g/mL, respectively, after HeLa cells treatment for a certain time of 12 h, 24 h, 48 h, and 72 h. At the concentration of 1000 *μ*g/mL, the inhibitory rate of Eca-109 cell was 69.71%. PKCF exhibited significant proliferation inhibition effect on Eca-109 cells.

#### 3.3.4. Effect on Proliferation of H22 Cells

**Figure 5:**
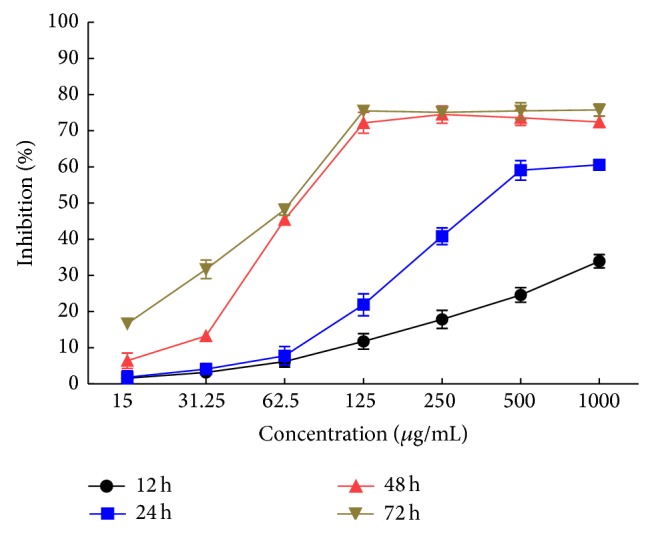
Time curve of different concentrations of PKCF against H22 cells proliferation.

As shown in Figure 5, PKCF inhibited the growth of H22 cell in a dose- and time-dependent manner with the IC_50_ values of 2505 ± 0.03940 *μ*g/mL, 445 ± 0.09288 *μ*g/mL, 98.36 ± 0.1703 *μ*g/mL, 70.96 ± 0.1082 *μ*g/mL, and 70.96 ± 0.05409 *μ*g/mL, respectively, after H22 cells treatment for a certain time of 12 h, 24 h, 48 h, and 72 h, and the inhibitory rate reached 78% till 72 h. Inhibition of PKCF on H22 cell proliferation was weaker compared with positive drug 5-fluorouracil (5-FU) at an inhibitory rate of 71% under the concentration of 1.56 *μ*g/mL and stronger than that of cyclophosphamide whose inhibition rate was of 62% after incubation for 68 h under the condition of concentration 500 *μ*g/mL while that of PCPC was 61% after incubation for 24 h under the same concentration [39].

#### 3.3.5. Effect on Proliferation of Vero Cells

**Figure 6:**
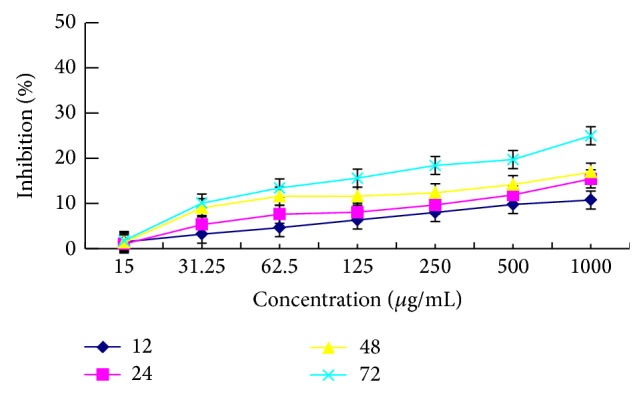
Time curve of different concentrations of PKCF against Vero cells proliferation.

As shown in Figure 6, the inhibitory effect of PKCF on Vero cells has a slight increasing trend with the increase of the concentration and time, and the inhibition rate was only up to 24.97% after incubation for 72 h at a concentration of 1000 *μ*g/mL. These observations illustrated that PKCF had less cytotoxic effect on the normal Vero cells.

In summary, the IC_50_ values regarding the growth of the cancer cells presented above demonstrate that PKCF has antitumor activity. Moreover, PKCF can significantly suppress the proliferation of H22 cells in vitro when compared to those of HeLa and Eca-109. This is the first report that PKCF can inhibit H22 cells growth in vitro, suggesting a potential therapeutic role of PKCF in the treatment of liver cancer. Further studies on the mechanism of PKCF inhibition of H22 cells remain to be conducted.

### 3.4. Antiaging Effects

Oxidative damage to macromolecules tends to accumulate in the cell with increasing age and is thought to be one of the causative factors of aging [40, 41].

#### 3.4.1. Effect on Lifespan of* Drosophila melanogaster*

Results of multiple comparison (Table 6) showed that the average lifespan and the average maximum lifespan of male* Drosophila* group at the dose of 0.0067% are significantly longer than those of control group and the other four groups (*P* < 0.01), and the rate of life extension was 15.49% and 10.43%, respectively. The average lifespan and the average maximum lifespan of female* Drosophila* groups at the dosage of 0.0067% and 0.0201% were significantly longer than those of control group (*P* < 0.01), and the rate of life extension was 16.77% and 14.03% and 13.86% and 8.91%, respectively. The average maximum lifespan of female and male* Drosophila* group at all the tested dosages was longer than that of control group (*P* < 0.01). Among them, the average maximum lifespan of dosage 0.0067% showed significant difference from the other groups (*P* < 0.01). Thus, PKCF could prolong* Drosophila melanogaster's* lifespan.

#### 3.4.2. Effect on Biochemical Index of* Drosophila In Vivo*

The statistical results (Table 7) indicated that there were significant differences in the activity of SOD and the content of MDA between PKCF groups and control group. The activity of SOD in female and male* Drosophila* group was increased in a dose-dependent manner (*P* < 0.01) and reached the peak at the concentration of PKCF 0.0201%. On the contrary, the content of MDA in female and male* Drosophila* group was decreased in a dose-dependent manner (*P* < 0.01) and was the lowest at the concentration of PKCF 0.0201%. Results of multiple comparisons showed that the activity of SOD of the group with 0.0201% dosage was significantly higher than the other four groups while the content of MDA was significantly lower than the other four groups in the female fruit flies. The activity of SOD and the content of MDA in male* Drosophila* group at the concentration of 0.0201% differed significantly from those of control group (*P* < 0.01).

Combining with the survival test results, it was demonstrated that* Drosophila* longevity was related to a balanced state of antioxidant capacity and lipid peroxidation in vivo. Exogenous antioxidants may improve the ability of free radical scavenging activity and delay aging according to the free radical theory of oxidative damage [42]. In this study, the free radicals in fruit flies were reduced after feeding with PKCF, lipid peroxide chain reaction produced by free radical could be terminated, and thus the cell membrane integrity and normal cell metabolism were maintained. On the other hand, PKCF enhances the antioxidant enzymes activity in vivo, and free radicals are cleaned in a time-dependent manner; therefore, the balance between oxidation and antioxidation is kept, leading to lifespan extension in fruit flies.

It was reported that carotenoids prolonged the average lifespan of fruit flies of 12.4% for male and 7.2% for female, and that of average longest lifespan was 2.1% (male) and 1.7% (female), respectively [43]. Therefore, the effect of PKCF on antiaging in* Drosophila* was stronger than that of carotenoids. The mechanisms underlying delayed aging by PKCF in the flies remain to be further studied.

## 4. Conclusion

According to the results of this study, it was clearly indicated that PKCF had remarkable antioxidant activity in vitro and in vivo, as its capacity to scavenge free radical in vitro was stronger than that of vitamin C and it inhibited MDA formation while it enhanced the activities of SOD in mice in vivo. In addition, the result of MTT assay exhibited that PKCF had pronounced antitumor activity, which inhibited the growth of H22, Eca-109, and HeLa cancer cells at low concentrations with average IC_50_ values of 70.96 ± 0.05409 *μ*g/mL, 260.4 ± 0.06887 *μ*g/mL, and 113.3 ± 0.08062 *μ*g/mL, respectively. Furthermore, the test of survival of fruit flies showed that PKCF had antiaging effect on* Drosophila*; that is, the average maximum lifespan and the average lifespan in male and female flies were prolonged by PKCF. Additionally, PKCF improved SOD activity and lowered MDA content in* Drosophila.* Our study suggests that PKCF has a potent antioxidant activity and could be utilized as a novel natural antioxidant in food and therapeutics. Further studies on the structure, function, and mechanisms of action are in progress.

## Figures and Tables

**Table 1:** Weight change of mice before and after gastric perfusion.

Group	Number of samples	Gavage in a dosage/mg·kg^−1^·bw^−1^·d^−1^	Weight before gastric perfusion/g	Weight after gastric perfusion/g	Added value of weight/g
NC	10	—	24.09 ± 0.86	34.57 ± 1.57	10.48 ± 2.88
NC^+^	10	78	24.75 ± 0.79	34.84 ± 1.86	10.08 ± 1.66
PKCF/LD	10	50	23.85 ± 0.85	33.94 ± 1.86	10.09 ± 1.46
PKCF/MD	10	100	25.25 ± 1.78	35.21 ± 2.58	9.96 ± 1.18
PKCF/HD	10	200	23.42 ± 1.30	33.10 ± 3.14	9.67 ± 3.25

**Table 2:** The effects of PKCF on spleen index and thymus index of mice.

Group	NC	NC^+^	LD	MD	HD
Thymus index	0.06 ± 0.05	0.05 ± 0.07	0.04 ± 0.09	0.05 ± 0.02	0.04 ± 0.08
Spleen index	0.06 ± 0.03	0.05 ± 0.04	0.06 ± 0.06	0.04 ± 0.01	0.04 ± 0.04

**Table 3:** Values of SOD in the tissues and serum of mice.

Group	Superoxide dismutase (SOD)		
	Serum (U/mL)	Liver (U/mg prot.)	Kidney (U/mg prot.)
NC	181.66 ± 27.36	52.01 ± 40.63	144.19 ± 8.43
NC^+^	211.06 ± 52.98^**^	87.98 ± 39.22^*^	184.03 ± 12.33^**^
PKCF/LD	190.59 ± 20.44	82.42 ± 9.59^*^	176.85 ± 12.56^*^
PKCF/MD	198.71 ± 21.47^*^	98.87 ± 5.57^*^	181.95 ± 7.59^**^
PKCF/HD	213.74 ± 23.73^**^	107.96 ± 11.53^**^	182.28 ± 24.27^**^

Note: compare with control group: ^*^ *P* < 0.05, ^**^ *P* < 0.01.

**Table 4:** Values of MDA in the tissues and serum of mice.

Group	Malondialdehyde (MDA)		
	Serum (nmol/mL)	Liver (nmol/mg prot.)	Kidney (nmol/mg prot.)
NC	18.57 ± 29.69	10.64 ± 3.88	10.24 ± 5.61
NC^+^	6.98 ± 29.69^*^	6.48 ± 5.59^*^	6.98 ± 2.25^*^
PKCF/LD	9.68 ± 5.74	8.60 ± 5.99	7.49 ± 2.36
PKCF/MD	6.12 ± 3.28^*^	6.04 ± 3.23^*^	4.50 ± 2.16^*^
PKCF/HD	6.08 ± 3.15^**^	5.67 ± 1.68^**^	1.81 ± 0.89^**^

Note: compare with control group ^*^ *P* < 0.05, ^**^ *P* < 0.01.

**Table 5:** Values of T-AOC of tissues and serum from mice.

Group	Total antioxidant capacity (T-AOC)		
	Serum (mg/mL)	Liver (U/mg prot.)	Kidney (U/mg prot.)
NC	4.30 ± 2.66	1.28 ± 0.60	1.81 ± 0.89
NC^+^	7.95 ± 4.70^*^	2.60 ± 0.79^**^	6.98 ± 2.25^**^
PKCF/LD	3.31 ± 1.66	2.22 ± 0.79^*^	4.50 ± 2.16^*^
PKCF/MD	7.10 ± 3.18^*^	3.18 ± 1.61^**^	5.49 ± 2.36^**^
PKCF/HD	19.11 ± 21.44^**^	3.77 ± 1.30^**^	10.24 ± 5.61^**^

Note: compare with control group: ^*^ *P* < 0.05, ^**^ *P* < 0.01.

**Table 6:** Effect of PKCF on lifespan of *Drosophila melanogaster* (*x* ± *s*).

Sex	PKCF dose (%)	Number	LT_50_ (d)	Average lifespan (d) *x* ± *s*	Average maximum lifespan (d) *x* ± *s*	Average life extension rate (%)	Average maximum life extension rate (%)
Female	0.000	60	43	41.50 ± 7.75^b^	50.80 ± 1.69^c^	—	—
	0.022	60	44	41.90 ± 8.94^b^	53.40 ± 1.65^b^	0.96	5.12
	0.067	60	49	47.93 ± 6.50^a^	56.10 ± 1.10^a^	15.49	10.43
	0.201	60	46	43.70 ± 8.93^b^	53.20 ± 0.63^b^	5.30	4.72
	0.603	60	43	40.50 ± 9.12^b^	50.20 ± 1.13^c^	—	—

Male	0.000	60	36	34.28 ± 8.32^b^	44.90 ± 1.73^c^	—	—
	0.022	60	38	36.23 ± 8.36^ab^	46.30 ± 0.82^c^	5.69	3.12
	0.067	60	41	40.03 ± 9.30^a^	51.20 ± 1.32^a^	16.77	14.03
	0.201	60	41	39.03 ± 9.61^ab^	48.90 ± 1.29^b^	13.86	8.91
	0.603	60	38	35.38 ± 9.30^b^	46.20 ± 1.03^c^	3.21	2.90

Note: *P* < 0.01, significant relationships between groups represented by a, b, c, and so forth.

**Table 7:** Effects of PKCF on content of MDA and activity of SOD (*n* = 5, *x* ± *s*).

PKCF dose (%)	SOD (U/mg prot.)		MDA (nmol/mg prot.)	
	Female	Male	Female	Male
0.0000	33.51 ± 1.10^b^	27.26 ± 0.78^b^	1.34 ± 0.27	1.47 ± 0.17^a^
0.0022	35.12 ± 1.09^b^	29.31 ± 0.90^ab^	0.70 ± 0.05^b^	0.93 ± 0.10^b^
0.0067	34.74 ± 1.80^b^	31.88 ± 1.19^a^	0.58 ± 0.07^b^	0.66 ± 0.15^b^
0.0201	41.56 ± 1.02^a^	33.13 ± 1.25^a^	0.29 ± 0.01^c^	0.39 ± 0.00^bc^
0.0603	31.01 ± 2.48^bc^	30.97 ± 1.10^a^	0.36 ± 0.02^bc^	0.44 ± 0.05^bc^

Note: *P* < 0.01, significant relationships between groups represented by a, b, c, and so forth.
